# Search for SINE repeats in the rice genome using correlation-based position weight matrices

**DOI:** 10.1186/s12859-021-03977-0

**Published:** 2021-02-02

**Authors:** Yulia M. Suvorova, Anastasia M. Kamionskaya, Eugene V. Korotkov

**Affiliations:** Research Center of Biotechnology of the Russian Academy of Sciences, 60 let Oktjabrja pr-t, 7, bld. 1, Moscow, Russia

**Keywords:** Transposons, SINE, Distant similarity, PWM

## Abstract

**Background:**

Transposable elements (TEs) constitute a significant part of eukaryotic genomes. Short interspersed nuclear elements (SINEs) are non-autonomous TEs, which are widely represented in mammalian genomes and also found in plants. After insertion in a new position in the genome, TEs quickly accumulate mutations, which complicate their identification and annotation by modern bioinformatics methods. In this study, we searched for highly divergent SINE copies in the genome of rice (*Oryza sativa* subsp. *japonica*) using the Highly Divergent Repeat Search Method (HDRSM).

**Results:**

The HDRSM considers correlations of neighboring symbols to construct position weight matrix (PWM) for a SINE family, which is then used to perform a search for new copies. In order to evaluate the accuracy of the method and compare it with the RepeatMasker program, we generated a set of SINE copies containing nucleotide substitutions and indels and inserted them into an artificial chromosome for analysis. The HDRSM showed better results both in terms of the number of identified inserted repeats and the accuracy of determining their boundaries. A search for the copies of 39 SINE families in the rice genome produced 14,030 hits; among them, 5704 were not detected by RepeatMasker.

**Conclusions:**

The HDRSM could find divergent SINE copies, correctly determine their boundaries, and offer a high level of statistical significance. We also found that RepeatMasker is able to find relatively short copies of the SINE families with a higher level of similarity, while HDRSM is able to find more diverged copies. To obtain a comprehensive profile of SINE distribution in the genome, combined application of the HDRSM and RepeatMasker is recommended.

## Background

Transposable elements (TEs or transposons) are genetic elements that can move around the genome, create new copies and integrate into a new place in the genome. In the genome sequences, TEs copies represent dispersant repeats and occupy most of the eukaryotic genomes. In plants they can occupy up to 90% of the total genome length. For many years these parts of the genome were considered as “junk DNA”; however, recently TEs have been shown to possess functional activity [1, 2]. Based on the mechanism of transposition and chromosomal integration, TEs are classified into DNA transposons and retrotransposons which in turn are divided into those with and without long terminal repeats (LTR and non-LTR) [3]. In plant genomes, LTR retrotransposons are the most represented. The number of copies of non-LTR retrotransposons—long and short interspersed nuclear elements (LINEs and SINEs)—are not as high as those of LTRs.

SINEs are non-autonomous TEs that do not encode their own proteins but utilize those coded by LINEs [4]. In mammalian genomes, SINEs are wildly represented (mainly by the Alu and mammalian-wide interspersed repeat (MIR) families) and extensively studied [5, 6]. However, even in mammals, it has been shown that only a part of all existing SINEs have been identified by standard repeat detection methods [7]. For plant genomes which contain fewer, SINEs, there is currently no unified set of SINE consensuses. Thus, from 4 to 20 SINE families have been reported in the rice genome depending on the classification method [8–10]. In the work we used a set of SINE consensus sequences collected in the work [10] (further referred here as EDTA set).

Typically, SINEs consist of a tRNA head, a body (whose origin is not completely clear), and an A-rich tail [11] and their lengths vary from 100 to 600 nucleotides. After insertion, SINE copies quickly diverge (accumulate mutations) [9], which prevent further transposon activity and protect the cell from uncontrolled copying activity; incomplete (truncated) SINE copies are also common [11]. It is known that the probability of substitution differs among nucleotides and that full sequence replacement does not occur immediately; at first, a purine-pyrimidine copy, which has low similarity to the initial sequence, is generated. This observation has helped to identify new copies of MIR-like elements in many genomes [5]. It is also known that cells use different repression mechanisms, including DNA methylation, to prevent further transposon movements across the genome, which in turn causes more frequent C → T substitutions [12, 13]. The detection of these TE copies by modern bioinformatics methods is complicated because of a large number of substitutions and other mutations that occur in the copy after the insertion.

Bioinformatics methods to search for SINEs as well as other transposons can be divided into de-novo and library-based [14]. The de-novo methods include structure-based approaches that use sequence characteristics of the target TE (such as the tRNA part, A-rich tail, etc.) for search and classification; they can be applied to detect different types of repeats in newly sequenced genomes. Typically, structure-based methods reveal full-length well-preserved transposon copies; the examples are SINE-Finder [15] and SINE-Scan [16]. Other de-novo methods such as RECON [17] and RepeatScout [18] (later combined into the RepeatModeler pipeline [19]) exploit homology and repetitiveness in the examined genome.

The search performed by the library-based methods requires an initial sequence library usually constructed by the de-novo methods. RepeatMasker is the most widely used library-based method suitable for the identification of all repeat types, including SINE [20]. RepeatMasker utilizes libraries of consensus sequences, such as Repbase [21] or Dfam [22] (applicable to a few model genomes) or a user-specified library. However, although currently RepeatMasker is the standard program for repeat detection and masking and is included in many genome annotations pipelines, it may not be universally applicable because highly divergent repeat elements are difficult to identify using traditional alignment-based methods [7]. Thus, it has been shown that RepeatMasker does not detect all the copies present in a genome and that the resulting annotation may not be accurate [7, 23]. Other programs using the library comparison approach are Maskeraid [24], PLOTREP [25], and Greedier [26], as well as tools such as BLAST [27] and its analogues. It is worth noting that the ability of both types of methods (similarity-based or de novo) decreases with the increase in the relative age of the repeat family, as substitutions and other types of mutations tend to accumulate with time [7].

Elements of the known SINE families can be searched using Hidden Markov Models (HMMs), which are built on multiple sequence alignment of full-size elements of the same family. HMMs of some SINE families (Alu and MIR) constructed for several model organisms and stored in the Dfam database [22] can also be used by RepeatMasker to search for divergent copies of these repeats. A limitation of this approach is that the initial sample for HMM construction is created using BLAST or similar methods that do not consider correlation between neighboring nucleotides; as a result, the correlation properties of different copies can eliminate each other, which can greatly reduce the search potential of an HMM [28].

To overcome the described limitations, we used the Highly Divergent Repeat Search Method (HDRSM), which considers both sequence similarity and correlations of nucleotide pairs within the compared sequences. Previously, a similar method was used to search for frameshifts in protein-coding sequences [28]. In this work, we applied the HDRSM to identification of SINEs in the genome of rice (*Oryza sativa* subsp. *japonica*) and found highly divergent SINE copies that were missed by the RepeatMasker program. Our results indicate that the HDRSM makes it possible to detect statistically significant similarities among DNA sequences containing both indels and multiple nucleotide substitutions.

## Results

### Parameter optimization

In the HDRSM, the position weight matrix (PWM) used to perform a genome-wide search is constructed taking into account the correlation between neighboring symbols. At the stage of matrix construction, the HDRSM utilizes an important parameter *Kd*, which is responsible for correct determination of boundaries in the local alignment (see PWM construction).

**Fig. 1:**
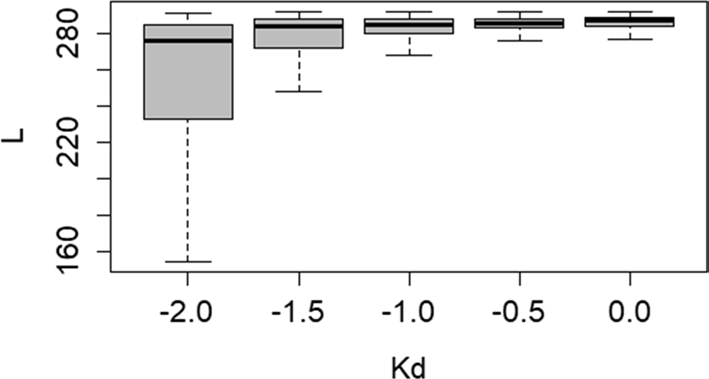
The dependence of the average local alignment length on *Kd* for the sequences from *FullLengthSet*

**Fig. 2:**
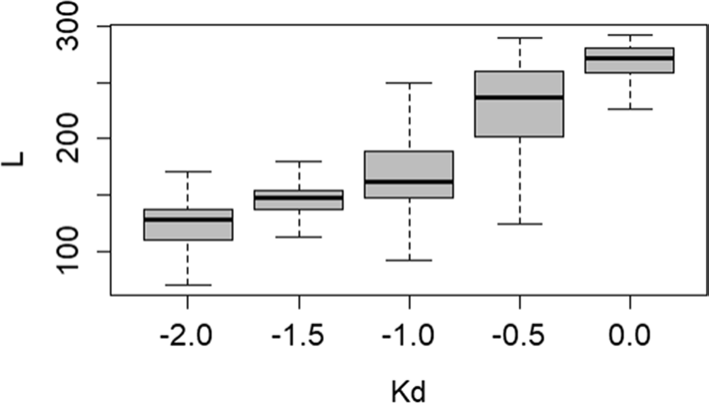
The dependence of the average local alignment length on *Kd* for the sequences from the *TrunkatedSet* set

To find an optimal *Kd* value that most accurately determines the boundaries of SINE copies, we performed a set of tests with artificial chromosomes containing insertions of mutated full-length copies of the OsSN1 consensus sequence (*FullLengthSet*) as well as truncated (*TrunkatedSet*) copies of OsSN1 (see Genome scanning procedure for the details of simulated set creation) using *K*_*d*_ values of 0.0, − 0.5, − 1.0, − 1.5, and − 2.0. First, a PWM for the full-length OsSN1 consensus sequence was constructed; then, it was transformed by applying the corresponding *Kd* value and used to perform a search for copies in the sequences from *FullLengthSet* and *TrunkatedSet*. Then, we calculated the average length and variance of the identified similarities for all tests within the corresponding set (Figs. 1, 2).

Figure 1 shows the search results for full-length mutated copies (*Full LengthSet*), which indicated that the decrease in *K*_*d*_ was correlated with the decrease in the average size of the identified sequences, which was smaller than the actual size of the inserted sequences (293 nt), Whereas Fig. 2 shows that for *K*_*d*_ > − 1.0, the length of found regions identified among truncated insertions (*TrunkatedSet*) was greater than the actual insertion size (150 nt). Thus, the program expanded the constructed local alignment by joining random fragments at the beginning and the end of the alignment, which means that for *K*_*d*_ > − 1.0, the detection of local alignment boundaries in sequences from *TrunkatedSet* is incorrect. As for *K*_*d*_ = − 1.0, the length of the fragments identified in both test sets (*FullLengthSet* and *TrunkatedSet)* was the closest to those of the originally inserted sequences, regardless of the number of substitutions; therefore, this *K*_*d*_ value was used in further analyses.

### SINE consensus set

**Fig. 3:**
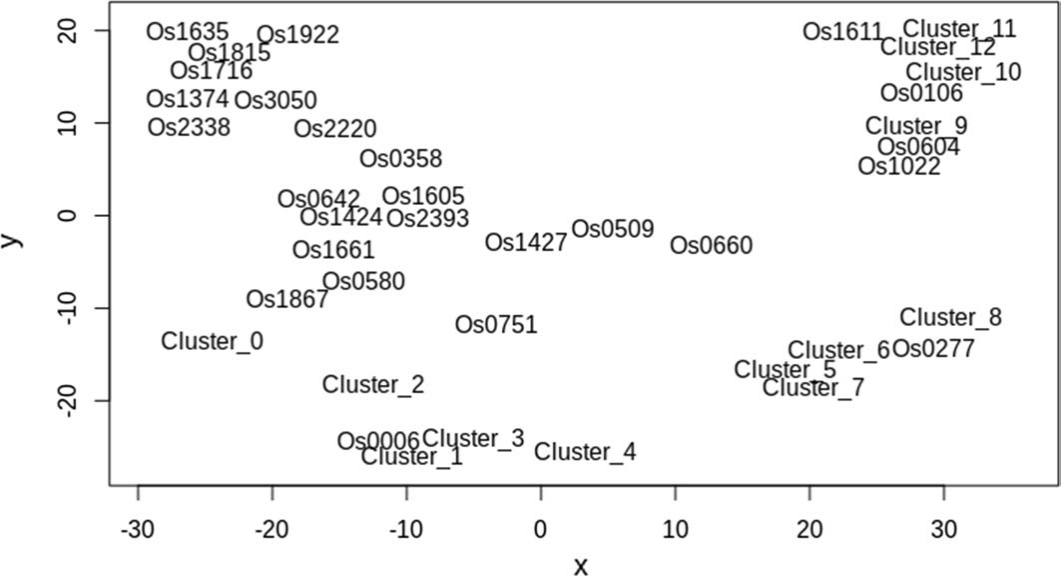
Two-dimensional representation of the relative distance between the consensuses in the EDTA set. The figure obtained in the result of multidimensional scaling analysis of the set (based on pairwise sequence identity)

In this work, we used a set of SINE consensus sequences referred as the EDTA set, which in turn consisted of two sets: one was collected using RECON [10] and the other containing 13 consensuses was constructed using SINE-scan [16]. We excluded sequences longer that 600 nt, and our dataset consisted of 39 sequences. The length of SINE consensuses in dataset varied from 85 nt (Os1611) to 516 nt (Os1815) with the mean about 280 nt. The identity between two consensuses in the EDTA set varied from 12.0 (between Os1611 and Os1815) to 97.3 (between Cluster_9 and Os0604) with the mean value of 38.96 (see Distance between EDTA set consensuses). The relative distance between consensuses in the EDTA set in two-dimensional space is presented in Fig. 3.

### Search for artificial SINE insertions

**Fig. 4:**
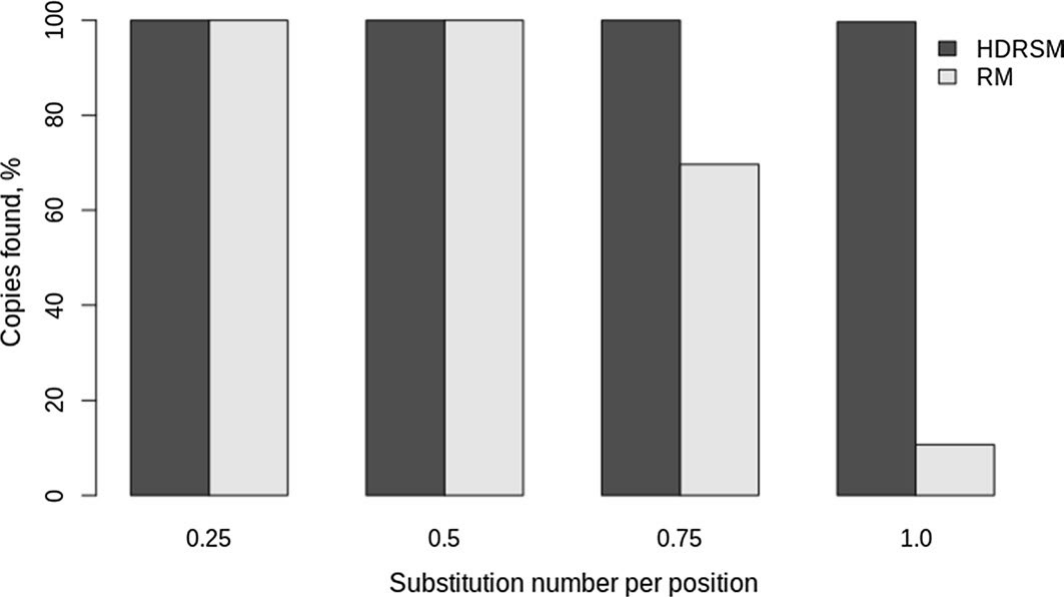
The percentage of copies found by the HDRSM and RepeatMasker programs on the test sequences from the FullLengthSet set, depending on the number of substitutions

**Fig. 5:**
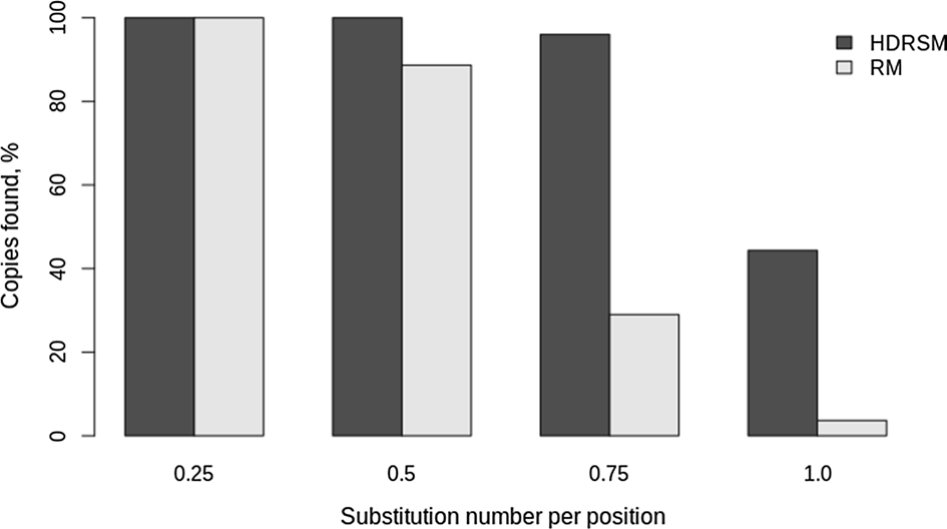
The percentage of copies found by HDRSM and RepeatMasker on the test sequences of the TrunkatedSet set depending on the number of substitutions made

Artificial chromosomes containing insertions of mutated OsSN1 copies from *FullLengthSet* and *TrunkatedSet* were scanned by the HDRSM (*Kd* = − 1.0) and RepeatMasker programs (see Genome scanning procedure). The complete OsSN1 consensus sequence was used as a library for all tests with both programs. After scanning artificial chromosomes from *FullLengthSet*, HDRSM detected all inserted copies (the data is shown in Fig. 4). While RepeatMasker found 100% insertions with 0.25 and 0.5 random substitutions per position and only 70% and 11% of insertions with 0.75 and 1.0 substitutions per position, respectively. In *TrunkatedSet*, the HDRSM detected 100%, 100%, 96%, and 44% and RepeatMasker—100%, 89%, 29%, and 4% of the inserted copies with 0.25, 0.5, 0.75, and 1.0 random substitutions per position, respectively are shown in Fig. 5. The results indicated that the HDRSM method could identify more divergent copies of SINEs than RepeatMasker, including both full-length and truncated copies.

**Fig. 6:**
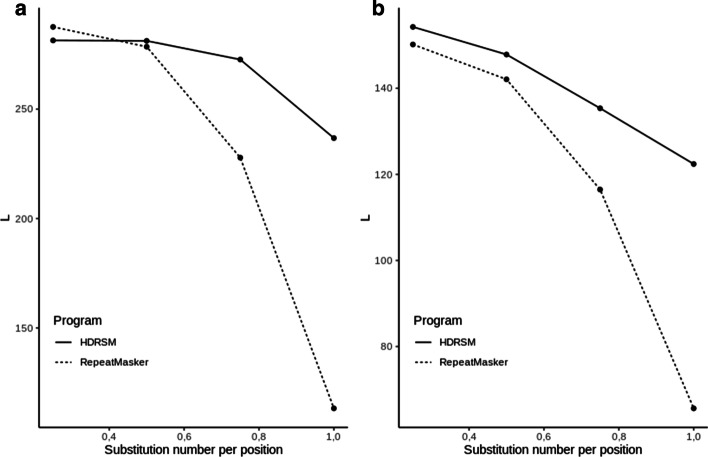
Change in the average length of the found copies when changing the number of mutations for full-length and truncated insertions of the SINE element

Figure 6a, b shows the dependency of the average length of the detected copies on the rate of substitutions for both methods. The data indicated that the HDRSM could more correctly define the boundaries of inserted copies, whereas RepeatMasker tended to find sequences shorter than the actual insertion. Furthermore, the larger was the number of substitutions inserted in the copy, the shorter was the corresponding fragment identified by RepeatMasker.

We measured false positives in the performed tests. We considered copies found outside the insertion region as false positive. Both methods produced 2–5 false positive hits per artificial chromosome.

### Classification test

To examine the ability of the programs to detect and correctly classify copies, we inserted divergent copies of pairs of consensuses into a shuffled chromosome. In the test, we used the most similar, and the most distant pairs of consensuses from the EDTA set and the pair with the middle value of identity; the tests were performed at 0.25 and 0.5 substitutions per position. In RepeatMasker, the classification procedure is embedded in the main program, whereas the HDRSM was applied separately to test chromosomes with each consensus; between the overlapping copies, we chose the one with the highest score.

The results of the tests are presented in Table 1. Both methods could identify and correctly classify almost all copies in the tests with the 0.25 substitution level, even in case of very similar sequences (Os604 and Cluster9) differing only in 5 positions (the HDRSM misclassified two copies in the last test). In the tests with the 0.5 substitution level, RepeatMasker correctly classified most of the identified copies (4% of misclassified copies between Os604 and Cluster_9) but could not detect all the copies, missing about 40% in some tests; at the same time, the HDRSM detected more copies, although in the test with the most similar consensuses up to 18% of the found copies were misclassified. The lower specificity is a consequence of the higher sensitivity of the HDRSM.

**Table 1:** The results of the classification test

	Os1611	Os1815	Os1611	Os0604	Os0604	Cluster_9
	*Substitution level—0.25*					
HDRSM	100/0	100/0	100/0	100/0	100/0,003	100/0,006
RepeatMasker	98/0	100/0	98/0	100/0	100/0	100/0
	*Substitution level—0.5*					
HDRSM	85/0	100/0	91/0	100/0	97/18	82/13
RepeatMasker	58/0	100/0	61/0	85/0	85/4	77/4

The table contains percent of correctly detected/misclassified copies for both method in the two consensus tests with 0.25 and 0.5 level of substitutions in the inserted copies

### Results of the rice genome analysis

Using the HDRSM, we constructed PWMs for each of the 39 consensus sequences from the dataset [10]. Then each PWM was transformed, and the value *K*_d_ = − 1.0 was used (see the "Methods" section). The obtained PWMs were then used to scan 12 chromosomes of the rice genome. As a result, more than 40,415 copies of the 39 examined SINE families were found.

To determine the percentage of false positives for the HDRSM method, nucleotides within each of the sequences of the 12 rice chromosomes were randomly shuffled. The shuffled chromosomes were processed using the 39 obtained PWMs. In total, 1156 copies with the Z value exceeding the selected threshold were identified by the HDRSM on the shuffled chromosomes.

Some consensus sequences from the EDTA set were highly similar and, therefore, the results of genome scanning were overlapping between families. To remove the redundancy associated with the similarity of consensuses, we performed the following selection procedure: if copies of different families overlapped by more than 20%, only the sequence with the largest Z value was included in the final sample. As a result, 18,117 copies of SINE repeats remained in the rice genome. The high number of intersections is associated with the similarity of the consensus sequences; furthermore, the HDRSM considered the correlation of neighboring nucleotides, which allowed recognition of distant similarities between families.

Low-complexity sequences can affect the results obtained by the program and correlations of symbols inside them can lead to the detection of false similarities. To filter out such sequences, we used the DUST program [29]. The copies with low-complexity regions constituting more than 10% of the total length were excluded from further analysis. This threshold was set based on the assumption that, although SINEs usually contain low-complexity sequences in their structure (the A-rich tail), their abundant presence (over 10% of the copy) most likely indicates an artifact. After excluding copies with low-complexity regions, 14 030 sequences remained. The numbers of copies for each SINE examined in the final sample are presented in the second column of Table 2.

**Table 2:** The statistics of the SINE copies found in the rice genome by the programs *HDRSM* and RepeatMasker

(1) Family name	(2) HDRSM	(3) RM	HDRSM			RM		
			(4) Overlap, same family	(5) Overlap, other family	(6) Not found	(7) Overlap, same family	(8) Overlap, other family	(9) Not found
Os0006	854	323	69	668	117	69	121	133
Os0106	716	945	648	10	58	649	241	55
Os0277	261	133	17	157	87	17	59	57
Os0358	76	220	18	0	58	18	0	202
Os0509	418	407	281	0	137	281	2	124
Os0580	155	104	19	75	61	20	13	71
Os0604	55	53	24	7	24	25	14	14
Os0642	201	136	22	17	162	22	26	88
Os0660	171	179	51	1	119	51	2	126
Os0751	942	1292	641	10	291	648	30	614
Os1022	160	135	80	4	76	81	4	50
Os1374	507	364	139	40	328	139	32	193
Os1424	188	141	32	31	125	32	20	89
Os1427	144	131	98	0	46	98	1	32
Os1605	54	37	10	0	44	10	0	27
Os1611	86	91	63	2	21	63	1	27
Os1635	372	511	216	2	154	217	0	294
Os1661	55	75	15	7	33	15	16	44
Os1716	613	401	222	4	387	245	4	152
Os1815	1441	458	225	1	1215	230	2	226
Os1867	152	70	14	79	59	14	5	51
Os1922	391	289	92	2	297	92	0	197
Os2220	493	524	193	17	283	195	57	272
Os2338	325	351	46	52	227	48	15	288
Os2393	272	297	42	42	188	42	43	212
Os3050	329	284	115	3	211	127	0	157
RST-Osativa-Cluster_0	380	78	14	297	69	14	51	13
RST-Osativa-Cluster_1	137	291	6	98	33	6	259	26
RST-Osativa-Cluster_10	527	648	395	11	121	397	141	110
RST-Osativa-Cluster_11	174	21	4	137	33	4	3	14
RST-Osativa-Cluster_12	299	30	14	237	48	15	9	6
RST-Osativa-Cluster_2	1676	2991	1302	91	283	1359	1039	593
RST-Osativa-Cluster_3	305	117	9	249	47	9	87	21
RST-Osativa-Cluster_4	209	189	7	162	40	7	136	46
RST-Osativa-Cluster_5	147	331	35	73	39	35	232	64
RST-Osativa-Cluster_6	235	201	51	129	55	52	99	50
RST-Osativa-Cluster_7	241	323	50	139	52	50	206	67
RST-Osativa-Cluster_8	238	102	8	175	55	8	45	49
RST-Osativa-Cluster_9	31	29	0	10	21	0	8	21
Total	14,030	13,302	5287	3039	5704	5404	3023	4875

Column 1—family name; Column 2—the number of copies of the family found by HDRSM; Column 3—the number of copies of the family found by RepeatMasker; Column 4—the number of copies of the family found by HDRSM that intersected with the result of RepeatMasker, and family names match; Column 5—the number of copies of the family found by HDRSM that intersected with RepeatMasker, but the family names do not match; Column 6—the number of copies of the family found by HDRSM but missed by RepeatMasker; Column 7—the number of copies of the family found by RepeatMasker that intersected with the HDRSM, and family names match; Column 8—the number of copies of the family found by RepeatMasker that intersected with the HDRSM, but the family names do not match; Column 9—number of copies of the family found by RepeatMasker but missed by HDRSM

### Comparison with RepeatMasker

The consensus sequences from the EDTA set were assembled into a single fasta file and transferred to the RepeatMasker program as a library. A threshold level lower than default settings (“-cutoff 160” option) was used for RepeatMasker, so that the results were comparable with those of the HDRSM based on the number of false positives. With the defined threshold level, RepeatMasker found 16,421 copies of 39 studied SINEs families in the rice genome and 1464 SINE copies in the randomly shuffled rice genome. In case of overlapping copies, RepeatMasker assigns an appropriate family inside the main procedure [30]; however, there could still be overlapping similarities in the results. Therefore, we also excluded cases with overlapping of more than 20% length; consequently, 16,021 copies remained. Furthermore, we applied the DUST program to exclude copies containing more than 10% low-complexity sequences, which resulted in 13,302 copies. The number of copies found for each SINE family by RepeatMasker is shown in Table 2 (column 3).

Next, we compared the coordinates of SINE family copies identified by the HDRSM and RepeatMasker. Since the procedure for assigning the copy to a family with a high level of similarity with another family may differ between the programs, we compared not only the coordinates of the copies assigned to the same family but also those of the copies assigned to other families. Of the total number of copies identified by the HDRSM, the coordinates of 5287coincided with the results of RepeatMasker for the corresponding families (Table 2, column 4); for RepeatMasker, the number of coincidences with the HDRSM was 5404 (Table 2, column 7). The reason for the difference is that in some cases, RepeatMasker split one copy found by HDRSM into two. A total of 3039 copies of SINE repeats found by the HDRSM matched the results obtained by RepeatMasker, but the latter assigned them to other families (Table 2, column 5); for RepeatMasker, this number was 3023 (Table 2, column 8). These results suggest that the programs differently classified the same sequences with high similarity to several consensuses. There were 5704 copies found by the HDRSM but not by RepeatMasker (Table 2, column 6), and 4875 copies found by RepeatMasker but not by the HDRSM (Table 2, column 9). Table 2 presents the detailed statistics for each SINE family.

**Fig. 7:**
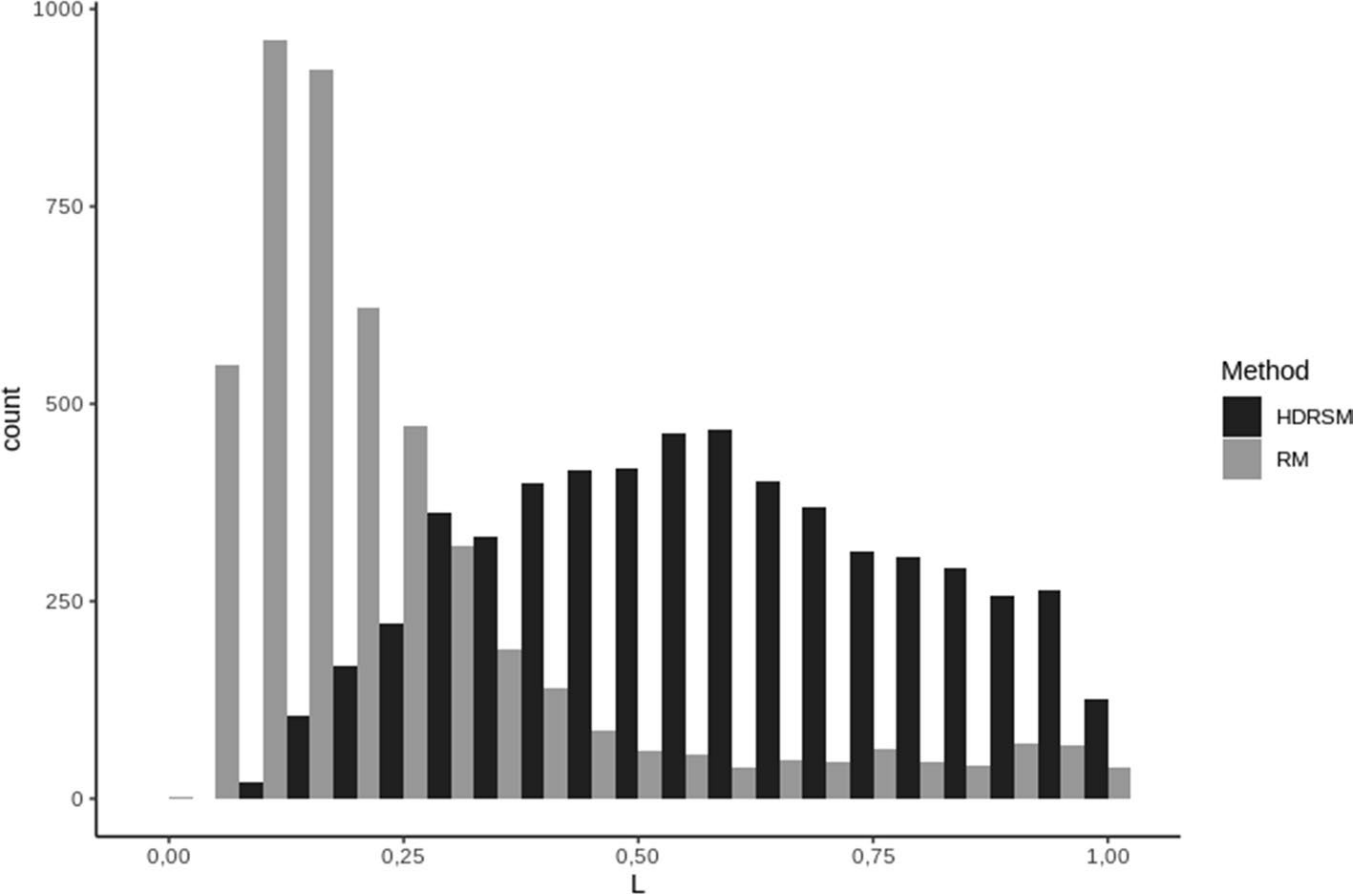
Distribution of percent of the lengths of unique copies relative to the length of the corresponding consensus found by RepeatMasker and missed by HDRSM and copies found by HDRSM and missed by RepeatMasker

Most of the copies found by RepeatMasker were shorter compared to those found by the HDRSM, which confirmed the results of the performed simulations. For the unique copies found by the methods (5704 copies found by the HDRSM and missed by RepeatMasker and 4875 copies found by RepeatMasker but not by the HDRSM) we constructed the distribution of the length of the found copy divided by the length of the corresponding family consensus (Fig. 7). It can be seen that unique copies found by RepeatMasker are mostly the shorter part of the corresponding consensus (less than 25%), whereas unique copies found by the HDRSM have longer size and constitute about 50% from the length of the corresponding consensus.

To investigate why some copies identified by RepeatMasker were missed by the HDRSM, we examined the ability of the programs to find consensus fragments of different lengths with a relatively low substitution level (0.25). In this test, we inserted fragments constituting 75%, 50%, 25%, and 17% of the OsSN1 length and carrying 0.25 substitutions per position (300 copies per each size) into shuffled chromosomes. The results indicated that for shorter fragments (< 50% of the consensus length), RepeatMasker outperformed the HDRSM (Table 3).

**Table 3:** The results of the test with short-part insertions

Length of inserted copies	HDRSM (%)	RepeatMasker (%)
220 nt (75% of consensus)	100	100
150 nt (50% of consensus)	100	100
75 nt (25% of consensus)	97.7	100
50 nt (17% of consensus)	83.3	90.3

The table contains the percent of copies found by each method for different length of inserted copies

## Discussion

It is important to analyze the difference between our approach and the methods used in RepeatMasker [30]. RepeatMasker uses the search for similar sequences when searching for dispersed repeats, but does not take into account the correlation existing between neighboring symbols. Considering the correlation component in the HDRSM allows us increase the statistical significance of the identified similarities, which is illustrated in the following example. Consider a DNA sequence “aattaaccaattaattccttccggggaaggaaggttccgg” in which symbols at positions k = 2, 4, 6, …, 40 are completely dependent on those at positions k = 1, 3, 5, …, 39. Assuming that a genome has the identical 40-nt sequence, we will evaluate the statistical significance of identifying this region considering only the similarity of individual bases and pairs of symbols and using the normal approximation to the binomial distribution. For the similarity of individual symbols, there will be a total of 40 matches. We estimate the probabilities of individual bases as p(a) = 12/40, p(t) = 10/40, p(c) = 8/40, and p(g) = 10/40; in this case, the probability of a match is *P*_1_ = 0.255. The average number of matching bases for the shuffled sequences is 10.2 with standard deviation of $\sqrt {10.2*0.745} \approx 2.76$; then, the argument of the normal distribution is *x*_1_ = (40–10.2)/2.76 ≈ 10.8.

Now let us calculate the argument of the normal distribution *x*_2_ when we compare the sequences using pairs of neighboring symbols. There are a total of 20 pairs without intersections. The probability of matching two identical pairs is (0.255)^2^ ≈ 0.065, the expected number of matching pairs is 1.3, and the standard deviation is $\sqrt {1.3*0.935} \approx 1,1$; therefore, *x*_2_ = (20–1.3)/1.1 = 17.0. From these calculations, it can be seen that *x*_2_ is more than 15 times greater than *x*_1_, indicating that the statistical significance of the match between two sequences calculated based on the correlation of nucleotide pairs is significantly higher than that calculated ignoring such correlation. For real sequences, *x*_1_ may be less and *x*_2_ may be greater than some threshold level *Z*_0_ (see Distance between EDTA set consensuses). Thus, taking into account the correlation factor makes it possible to detect more statistically significant similarities. As can be seen in Local alignment of a DNA sequence with the PWM, the HDRSM performs sequence alignments considering the correlation of neighboring symbols, whereas RepeatMasker finds alignments without it. In our opinion, this factor allows the HDRSM identify more copies than RepeatMasker with a higher level of statistical significance.

We have also compared HDRSM with the HMM-based method. Since there are no models for SINE elements in rice, we used the one from the human genome. We chose HMM created for a MIR family from the Dfam database (DF0000001.4). We implemented the nhmmer program [31] (HMMER-3.3. package, default mode, E-value = 10.0) to search for copies of DF0000001.4 in chromosome 22 of the human genome. In the result, nhmmer found 10,116 copies. Then we used the set of SINE sequences included in the model DF0000001.4 to create correlation based PWM for our method and analyzed human chromosome 22 as well as a randomly shuffled versions of the chromosome. HDRSM found 13,478 copies with the same rate of false positives (up to ten false positives per shuffled chromosome). The comparison of the coordinates of the copies found by nhmmer and HDRSM showed that 7963 copies were found by both methods. The results indicate that when we have a set of sequences instead of one consensus HDRSM may show even better results. We have studied the length of the copies that were found by nhmmer but missed by HDRSM. Most of these copies (75%) are shorter than 100 nt, the average length is about 65 nt (the length of the original repeat is 262 nt), while the average length of the sequences that were found by HDRSM but misses by nhmmer is about 120 nt. We can assume that nhmmer like RepeatMasker, can miss relatively long but highly divergent sequences and HDRSM, can miss short, truncated copies.

## Conclusions

In this study, we performed a search for highly divergent copies of SINE repeats in the rice genome using the HDRSM method, which considers symbol correlations within the sequence during PWM construction and further scanning and compared its performance with that of RepeatMasker. The developed method was tested and applied to search for more divergent copies of SINE repeats in the rice genome. Among the 15,423 detected copies of 39 SINE families 5704 were missed by RepeatMasker and 4875 copies missed by HDRSM. RepeatMasker could identify relatively short SINE copies with a high level of similarity, whereas the HDRSM was able to find longer and highly divergent copies; furthermore, RepeatMasker was prone to crop copies. The results indicate that to obtain a complete picture of SINE distribution in a genome, simultaneous use of the HDRSM and RepeatMasker is recommended.

The function of both the HDRSM and RepeatMasker depends on a set of consensus sequences. Therefore, it is important to develop an accurate open-source consensus database for different plant species. Currently, researchers can choose from several consensus sets, or de-novo create their own library and apply the HDRSM, which would help to identify highly divergent SINE copies. In the absence of a comprehensive consensus database, the HDRSM could be used with only a few SINE copies as a library.

It is important to note that the HDRSM is universal and can be applied to search for highly divergent copies of repeat types other than SINEs. For this, appropriate consensus sequences should be available to construct an initial PWM, which then can be used to screen the genome for copies of various repeats.

## Methods

In this work, we used the HDRSM which compares the PWM and a genome fragment using a modified dynamic programming procedure. The modification consists in considering the correlation of neighboring nucleotides within the sequence along with the similarity between the PWM and the target sequence; these correlations are also taken into account while building the PWM. The HDRSM includes three main steps—PWM construction, genome scanning based on the obtained PWM, and identification of significant similarities. Each of these steps is described in detail below.

### PWM construction

The PWM was created for a SINE family represented by a consensus sequence *S* of length *N*. The number of columns in such matrix was ​​*N* − 1 and the number of rows was 16, since we considered pairs of adjacent symbols at positions *k* − 1 and *k*, which allowed for correlation of neighboring symbols in the matrix. The PWM denoted as *M* (*l*, *k*) (*l* ranging from 1 to 16 and *k*—from 2 to *N*) was calculated by elements as:

1 $$m(l,k) = \frac{1.0 - f(i,j)}{{\sqrt {f(i,j)(1 - f(i,j))} }}$$

where *l* = *i* + 4(*j* − 1), and *i* and *j* are nucleotides in positions *k* − 1 and *k* of *S,* respectively.

Since we used only one consensus sequence for the family, the remaining 15 *m*(*l*,*k*) values were equal to zero. The first PWM column, *m*(*l*,1), was set as 1, and the values of the first column were used in Local alignment of a DNA sequence with the PWM.

Next, the obtained PWM *M*(*l*,*k*) was transformed to keep the following parameters constant:

2 $$R^{2} = \sum\limits_{l = 1}^{16} {\sum\limits_{k = 2}^{N} m } (l,k)^{2}$$

3 $$K_{d} = \sum\limits_{l = 1}^{16} {\sum\limits_{k = 2}^{N} m } (l,k)p_{1} (l)p_{2} (k)$$

where *p*_2_(*k*) = 1/N − 1, *p*_1_(*l*) = *p*(*i*)*p*(*j*), and *p*(*i*) and *p*(*j*) are probabilities of *i* and *j* nucleotides in *S*: *i,j* $\in${a,t,c,g}. The matrix transformation procedure was described previously [32].

The transformation was aimed to obtain the same *K*_*d*_ value for matrices with different numbers of columns constructed for sequences of different lengths. *K*_*d*_ is the equivalent of an expected *E* score value [33], which defines the accuracy of determining the start and end of the local alignment. If *Kd* ≤ − 1.5, then shorter alignments would take precedence over longer ones, and if *K*_*d*_ is about zero, then almost all local alignments would have a length equal to N. The optimal *K*_*d*_ value was chosen using simulations of SINE insertions in the genome.

### Local alignment of a DNA sequence with the PWM

The local alignment procedure was modified to account for the correlation of neighboring nucleotides. In the alignment, two sequences were considered: *S*_1_, which is a part of the analyzed genome of length *N*, and *S*_2_, which is a numerical sequence “1,2, …,* N*”; *S*_1_ and *S*_2_ are denoted as *s*_1_(*i*), and *s*_2_(*i*), respectively (where *i* is 1 to *N*). Then, sequence *S*_1_ was aligned with *S*_2_ using PWM *m*(*i*, *j*), where *i* and *j* range from 1 to 16 and from 2 to N, respectively. The *F* score was calculated using the following equations:

4 $$\begin{gathered} F(i,j) = \max \left\{ \begin{gathered} 0 \hfill \\ F(i - 1,j - 1) + m(n,s_{2} (j)) \hfill \\ F_{x} (i - 1,j - 1) + m(n,s_{2} (j)) \hfill \\ F_{y} (i - 1,j - 1) + m(n,s_{2} (j)) \hfill \\ \end{gathered} \right\} \hfill \\ \hfill \\ \end{gathered}$$

where *n* = *s*_1_(*k*) + 4(*s*_1_(*i*)-1)); if *I* = 1 *n* = *s*_1_(1).

5 $$\begin{gathered} F_{x} (i,j) = \max \left\{ \begin{gathered} F(i - 1,j) - d \hfill \\ F_{x} (i - 1,j) - e \hfill \\ \end{gathered} \right\} \hfill \\ \hfill \\ \end{gathered}$$

6 $$\begin{gathered} F_{y} (i,j) = \max \left\{ \begin{gathered} F(i,j - 1) - d \hfill \\ F_{y} (i,j - 1) - e \hfill \\ \end{gathered} \right\} \hfill \\ \hfill \\ \end{gathered}$$

where *d* is gap open penalty and *e* is gap extension penalty; here, we used *d* = 32.0 and *e* = 8.0 (based on model sequences, the choice of penalty for gap opening and extension was discussed in detail in [32]; we set *F*(0,0) = 0 and *F*(*i*,0) = *F*(0,*i*) = 0).

Where *n* = *s*_1_(*k*) + 4(*s*_1_(*i*)-1)), *i,* and *j* run from 2 to *N.* If *i* = 1 *n* = *s*_1_(1). We introduced the *n* parameter to account for the correlation of the neighboring symbol in *S*_1_ when performing the alignment; to determine *n*, a previous symbol of sequence *S*_1_, which was already included in the alignment, should be found. An element *m*(*n*,*s*_2_(*i*)) is selected based on index *k* calculated from the traceback matrix filled at position (*i*, *j*). If the previous *S*_1_ symbol included in the alignment is *s*(*i* − *t*), then *k* = *i* − *t* and *n* = *s*_1_(*i* − *t*) + (*s*_1_(*i*) − 1)∙4 at *t* = 1, which corresponds to the movement along the main diagonal of matrix *F* and there is no deletion of the symbol in *S*_1_; if *t* > 1, it corresponds to a deletion of *t* − 1 symbols in sequence *S*_1_. Deletion can also occur in sequence *S*_2_, which corresponds to deletion of a column in matrix *M*. If the previous *S*_2_ symbol included in the alignment has the number (*j* − 1), then there is no deletion in *S*_2_; if it is *j* − *t* (*t* > 1), then there is deletion of *t* − 1 symbols in sequence *S*_2_. In case of such transitions, there are no correlations between adjacent symbols. If the number of deletions is not large, it does not affect the result. In this case, *n* = *s*_1_(*i*) and *s*_2_(*j*) = 1 in Eq. 6; the values ​​are taken from the first column of the matrix *M*.

The traceback matrix was filled along with the dynamic programming matrix *F*. In each cell (*i*, *j*) of the traceback matrix, we stored the number of the matrix *F*′ cell at which the maximum is reached, using Eqs. 2–6; then, from the position corresponding to the maximum *F* value (*F*_*max*_)*,* we deduced the local alignment of the sequences using the traceback matrix.

To estimate the statistical significance of the obtained alignment, we performed simulations by generating a set of random sequences through random shuffling of sequence *S*_1_ 200 times. Then, *F*_*max*_ corresponding to each shuffled sequence was calculated and used to estimate mean $\overline{{F_{\max } }}$ and variance *D*(*F*_*max*_). The statistical significance was calculated using the following equation:

7 $$Z = \{ F_{\max } - \overline{{F_{\max } }} \} /\sqrt {D(F_{\max } )}$$

### Distance between EDTA set consensuses

To explore the relationship between consensuses in the EDTA set, we analyzed the identity between each consensus pair. Pairwise global alignment was performed with a simple scoring scheme using R Biostrings package [34] and the identity based on the alignment was measured using PID1 formula from the same package: 100 × (identical positions)/(aligned positions + internal gap positions). The distance between two sequences was calculated as (100 − identity) and the 39 × 39 distance matrix was constructed. To visualize the distance between consensuses, we applied multidimensional scaling to the distance matrix using *cmdscale* function in R and then used wordcloud R library to obtain graphical presentation of the distance in a two-dimensional space.

### Genome scanning procedure

To search for SINE copies in the rice genome, we used a sliding window of length *N* (equal to the length of the consensus sequence) and assumed that the starting position of the window in the chromosome sequence was *k*. The window was moved along the chromosome with a step of 10 nt, and *Z*(*l*) was calculated for each position according to Eq. (7), where *l* = + int (*k*/10); Then, we moved the window by 10 nucleotides and again performed calculations of *Z(l*). The calculations were repeated until *k* = *L* −* N* + 1, where *L* is the length of the analyzed rice chromosome. Then, the local maximum in the numerical *Z* (*l*) series was selected as the value exceeding a threshold *Z*_0_, which was chosen based on the condition that the number of copies of a SINE family found in the randomly shuffled rice genome should be about 20 (false positive hits). Our simulations showed that this condition corresponded to *Z*_0_ = 10.0. For all local *Z*_*max*_ > 10.0, we calculated the coordinates and constructed the alignments designated as copies of the corresponding SINE families.

### Simulated datasets

To compare the ability of the HDRSM and RepeatMasker to detect copies with low similarity to the consensus as well as to correctly determine copy boundaries, we performed a set of tests simulating the presence of divergent SINE copies in a chromosome. The OsSN1 SINE from the *SineBase* database [9] was used as the original SINE sequence. The length of the sequence is 293 nucleotides.

To scan all test sequences and the rice genome RepeatMasker was run with the following parameters: -no_is -nolow -cutoff 160.

#### FullLengthSet tests

In this series, the full-length OsSN1 sequence (293 nt) was used. In the first test, the sequence was modified by introducing 0.25 substitutions per position and in the second, 0.5, 0.75, and 1.0 substitutions per position were made; in addition, each copy had 2–5 random indels. In total, 300 OsSN1 copies for each substitution level were created and inserted into a rice chromosome whose sequence was shuffled prior to insertions to remove traces of SINEs and other transposons that could be present. All modifications, insertions, and chromosome shuffling were performed in a random manner. Since the random number generator was used to choose the positions for substitutions, multiple substitutions at the same position are possible.

#### TrunkatedSet tests

In this series of tests, only the first 150 nt of OsSN1 were used as the initial sequence. Then, similarly to the first series, 0.25, 0.5, 0.75, and 1.0 substitutions per position and 2–5 indels were randomly introduced into the initial sequence, yielding 300 copies per substitution, which were randomly inserted into a shuffled rice chromosome.

#### Small part tests

Additional tests to examine the ability of the method to detect truncated SINE copies of different length was performed using OsSN1 fragments of 220, 150, 75, and 50 nt constituting 75%, 50%, 25%, and 17%, respectively, of the original OsSN1 length (293 nt); all fragments contained 0.25 substitutions per position. As in the other experiments, 300 mutated copies of each length were generated and inserted into a randomly shuffled chromosome.

#### Two consensus tests

The last set of tests was developed to explore the ability of the methods to distinguish between copies of different consensuses. In each of these tests we used copies of two consensuses. In the first test, two most similar consensuses (Cluster_9 and Os0604) were used. For each of them, we generated 300 copies with 0.25 substitutions per position and 2–3 indels per copy and inserted the resulted copies into a randomly shuffled chromosome, which was analyzed with RepeatMasker and HDRSM using Cluster_9 and Os0604 as the initial library. As a result, we analyzed the number of correctly discovered copies as well as that of misclassified copies. The same test was performed with the most distant consensus pairs Os1611/Os1815 (identity = 12.0) and the consensuses Os0604 and/Os1611 with Identity = 36.54. And another set of tests was performed with the same three pairs of consensuses (Cluster_9/Os0604, Os0604/Os1611, and Os0604/Os1611) but in this case the substitution level between the consensus and the inserted copies was 0.5 per position.

## Data Availability

Consensus sequences of the SINE families related to the rice genome were downloaded from EDTA database materials [10]—https://github.com/oushujun/EDTA. The rice genome sequences (Oryza sativa Japonica) were downloaded from the Ensembl/Plants database, along with the annotation in gff3 format. The RepeatMasker program was downloaded from http://www.repeatmasker.org/RMDownload.html. The coordinates of found SINE copies in the rice genome and the results of the tests (by both HDRSM and RepeatMasker programs) are available online on the webpage https://github.com/suvorovay/SINEsuppl along with simulated datasets (FullLengthSet—ttps://github.com/suvorovay/SINEsuppl/tree/master/FullLengthSet, TrunkatedSet—https://github.com/suvorovay/SINEsuppl/tree/master/TrunkatedSet). TwoConsensusSet—https://github.com/suvorovay/SINEsuppl/tree/master/2Consensus_test. The executable files (Windows OS) for PWM construction, genome scanning, and result transformation can be downloaded from the https://github.com/suvorovay/SINEsuppl/exe_files.

## Abbreviations

SINE: Short interspersed nuclear elements
LINE: Long interspersed nuclear elements
HDRSM: Highly divergent repeat search method
PWM: Position-weight matrix
TE: Transposable elements
